# Collimator rotation in volumetric modulated arc therapy for craniospinal irradiation and the dose distribution in the beam junction region

**DOI:** 10.1186/s13014-015-0544-z

**Published:** 2015-11-19

**Authors:** Qilin Li, Wendong Gu, Jinming Mu, Wenming Yin, Min Gao, Juncong Mo, Honglei Pei

**Affiliations:** Department of Radiation Oncology, The Third Affiliated Hospital of Soochow University, The First People’s Hospital of Changzhou City, 185 Ju Qian Jie, Changzhou City, 213003 Jiangsu Province China

**Keywords:** Craniospinal irradiation, Volumetric modulated arc therapy, Collimator rotation, Beam junction region

## Abstract

**Purpose:**

The purpose of this study was to investigate the role of beam collimator rotation in Volumetric Modulated Arc Therapy (VMAT) for craniospinal irradiation (CSI), and the impact on dose distribution in the beam junctions.

**Methods:**

Six adult patients were selected for the study. Six VMAT plans with different collimator angles were generated for each patient. The patients were treated in supine position with two beam isocenters. The plans were evaluated by analysis of Dose-Volume Histogram (DVHs) data for planning target volume (PTV) and organs at risk (OAR), and conformity index (CI) and homogeneity index (HI) for the target. Dose distributions in the beam junctions were examined carefully and experimentally validated in phantom, with measurement using an ion chamber array and film.

**Results:**

The mean values of HI and CI for the plans with different beam collimator angles were not significantly different. The numbers of segments, monitor units (MUs) and the delivery time of the plans with 45° beam collimator were obviously higher than those in plans with other beam collimator angles. When collimator angle for both sets of beams were set at 0°, there was a 1 mm low dose gap measured in the junction region.

**Conclusions:**

By setting the collimator angle to 45°, only two isocenters were needed for the treatment of a target with the length up to 90 cm. The HI and CI of the plans were almost the same, regardless if the collimator angles were at 0°. The collimator angles for at least one set of beams should be off 0° in order to avoid a dose gap in the beam junction region.

**Electronic supplementary material:**

The online version of this article (doi:10.1186/s13014-015-0544-z) contains supplementary material, which is available to authorized users.

## Background

Craniospinal irradiation (CSI) is a standard therapy for some primary central nervous system (CNS) tumors like medulloblastoma and ependymoma [1–5]. The traditional CSI techniques are complicated; the patients in prone position are uncomfortable thus with poor reproducibility, and with supine position it is rather difficult to verify the field junctions. In addition, the poor dose uniformity in the region of field junction is often unacceptable clinically. In recent years, various intensity modulated radiation therapy (IMRT) techniques have been used for CSI [2–4]. Helical tomotherapy can be a favorable treatment because it obviates the problem of field junction due to its nature of beam delivery [1, 6–8]. The dosimetric advantages of proton therapy were for CSI were also evaluated [9]. However, a special machine like a tomotherapy unit may not be accessible for all patients. Due to relatively low incidence of CSI cases, it is desirable to develop a robust treatment technique for CSI using a conventional linear accelerator with a multileaf collimator (MLC). Supine position is preferred for its superior reproducibility. The least sets of fields, or number of beam isocenters should be used as more field junctions would increase the time for patient setup and junction verification, bring additional uncertainty in the region of field junction, and possibly take longer beam-on time to treat.

In this study we investigated the volumetric modulated arc therapy (VMAT) technique for CSI incorporated with collimator rotation, in order to limit the number of field sets to two. A beam with 45° collimator angle can cover the longest for a tube-like shape of target such as the spine, as shown in Additional file 1: Figure S1.

A high-end digital linear accelerator Axesse® (Elekta AB Stockholm, Sweden) equipped with the Agility® 160-leaf MLC was used for patient treatment. The width of all leaves is 5 mm at the isocenter plane. A distinguished advantage for the Agility MLC is its low leaf and interleaf transmission. The average MLC transmission for 6MV photon is 0.47 % (Table 1), vs. 1–2 % for other commercial systems [10]. The impact on dose distribution in the field junction region with various collimator angles, or directions of MLC leaf travel was evaluated using accurate Monte-Carlo based dose calculation.

**Table 1:** Parameters of the Axesse® linacs

Parameter	Axesse®
Leaf width at isocenter (mm)	5
Field size, maximum (mm)	400
Leaf individual travel range (mm)	200 (with respect to DLG^a^)
Leaf and DLG^a^ or backup jaw combined travel range (mm)	350
Leaf interdigitation range (mm)	200
Diaphragm over-travel relative to central axis (mm)	120
Height of leaf (mm)	90
Leaf and interleaf transmissions (6MV)	0.47 %
Diaphragm/jaw speed, maximum (mm/s)	90
Leaf speed, maximum (mm/s)	35
Leaf and DLG combined speed, maximum	65 mm/s
Speed of gantry rotation (/s)	6.0 degree/s
Dose rate, maximum (MU/min)	Continuous, 660

^a^ *DLG* Dynamic Leaf Guides. All leaves were integrated with two DLGs and they traveled together

## Methods

This study was approved by the Institutional Review Board of our hospital, and written informed consents were obtained from the patients before treatment. Six adults (Four females, Two males) were treated with 6MV photon (Table 2). Patients were in supine position, immobilized in a BodyFIX® vacuum cushion from head to hip (Elekta AB, Stockholm, Sweden) with a headrest, a knee support, and a thermoplastic mask. The planning CT scan was acquired using a Siemens Somatom® Sensation Open 40-slice CT scanner (Siemens Medical Solutions, Forchheim, Germany). The slice thickness of CT images was 3 mm. The image set was exported to a Monaco Treatment Planning System (Monaco version 3.2, Elekta AB, Stockholm, Sweden) for planning.

**Table 2:** Patient characteristics, treatment plan design and quality assurance

Patients	
Age (years)	31–53
Positioning	Supine
Fixation	Thermoplastic mask, vacuum bag and knee support
Patient’s height (cm)	152–175.6
Plans	
Length of target (cm)	68.1–80.7
Height of target (cm)	13.1–15.6
Width of target (cm)	14.2–15.8
The number of isocenters	2
Distance between isocenters (cm)	35–41
Total dose prescription (Gy)/Fraction	36/20
Quality assurance	
Device	MatriXX plus MultiCube (IBA inc.) EBT2 film (International Special Products inc.)
Image guidance	Cone-beam CT, daily

### Target delineation and dose prescription

The first clinical target volume (CTV), labeled as CTV1, consisted of the whole brain, and the second CTV, CTV2 consisted of the entire spinal canal with lateral extensions to include part of the nerve roots. The planning target volumes (PTV), PTV1 and PTV2, were generated by uniformly expanding CTV1 and CTV2 with a 5 mm margin, respectively. The dose prescription was 36Gy in 20 fractions to at least 95 % of the total PTV which consisted of PTV1 and PTV2.

### Setup of arc beams

Two sets of beams were used for the PTV length in a range of 68.1 cm to 80.7 cm (Table 2). The two beam isocenters shared the same X and Z coordinates, as those of the center of PTV. The shift in Y coordinate (in the craniocaudal direction) was easily implemented with a couch slide.

The PTV2 was a cylindrical structure with 6-8 cm diameter for an adult patient. With a 25–30° collimator rotation a target of 42 cm length could be covered, and with 45° collimator rotation a target of up to 48.5 cm long could be covered, as shown in Additional file 1: Figure S1. By a conservative estimation, two abutting sets of arc beams with 45° collimator angle can cover a target for CSI as long as 90 cm, even considering the brain target of 20 cm width, and the curved spinal target. Three beam isocenters may be necessary when the length of the target is over 90 cm.

**Fig. 1:**
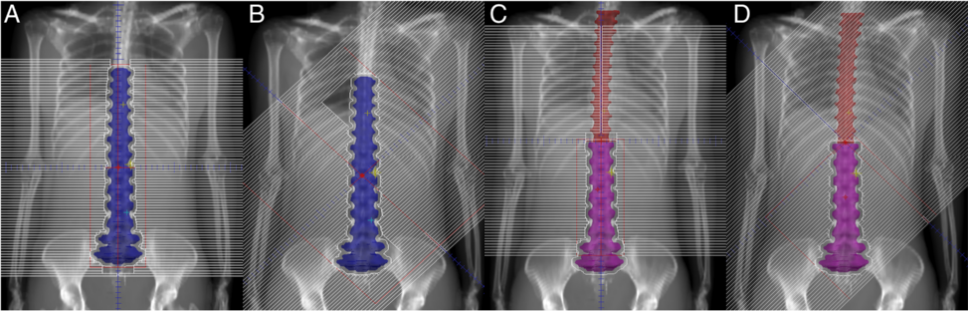
MLC conformed to a long target at 0° and 45° collimator angles. **a**, **b** collimator cannot conform well to the target of 36 cm long at 45°; **c**, **d** 48 cm long target divided into two parts of 24 cm in length; **d** the lower half of the target is conformed well with the collimator at 45°

It is important to recognize the limit of leaf interdigitation range of Agility® MLC, which is 20 cm. This means that the maximum distance between any two leaf ends on one side cannot not be larger than 20 cm. Thus the MLC cannot conform to a target shape longer than 28.2 cm with the collimator angle at 45° (Fig. 1). A solution to this problem is to divide the PTV into several parts. Each part has a length not greater than 24 cm. Arc beams are arranged to treat individually each part of the PTV. Therefore, only increased number of arc beams are used which involve shifts of MLC leaf guides, but not the number of beam isocenters.

### VMAT planning

A full arc beam with the upper isocenter covered mainly the head and neck part of the PTV. In order to reduce the volume of body irradiated, the lower set of beams irradiating the rest of the PTV consisted of 3 partial arcs: 180°–240°, 300°–60°, 120°–180°. This also led to better sparing of lung, liver and intestine than using the full arc, thereby reducing the toxicities of treatment.

An important issue to consider is how to match the beams with more than one isocenter to guarantee a smooth dose distribution in the junction region. A strategy that employs at least 2 cm overlapping region between beams with different isocenters was adopted [2–4]. The VMAT optimizer in the Monaco treatment planning system (TPS) would automatically produce a smooth dose distribution in the beam junction region, which was carefully checked and verified.

The plan quality potentially influenced by different beam collimator angles was investigated with different combinations of beam collimator angles in 0°, 30° and 45° for the upper and lower sets of beams. Six plans were generated for each patient which were named by PAc0c0, PAc0c30, PAc0c45, PAc30c30, PAc30c45, and PAc45c45. The detailed specification of these plans was given in Table 3.

**Table 3:** The specification of the treatment plans

	Beam collimator angle^a^		Number of arc beams
Plan name	Upper isocenter	Lower isocenter	
PAc0c0^b^	0°	0°	4
PAc0c30	0°	30°	4
PAc0c45	0°	45°	7^c^
PAc30c30	30°	30°	4
PAc30c45	30°	45°	7^c^
PAc45c45^b^	45°	45°	7^c^

^a^There were 2 beam isocenters in this study

^b^The patient whose PTV was 80.7 cm long was excluded for PAc0c0 and PAc45c45

^c^There were six partial arc beams with lower isocenter when there was 45 collimator angle. The target covered by the beams with the lower isocenter was divided into two parts then the beams conformed to them respectively

### Plan evaluation

The plan quality with respect to PTV coverage and dose received by organs at risk (OAR) was evaluated using the dose-volume histogram (DVH) data. For the PTV, the evaluated parameters were the mean dose, D_2_ and D_98_, or the dose delivered to 2 % and 98 % of the PTV, respectively, and V_107%_, or the volume in PTV receiving 107 % of the prescribed dose. A conformity index (CI) and a homogeneity index (HI) were calculate for each plan. CI and HI are defined as follows [11]:

$$ CI=\frac{Vo{l}_{Target}}{Vo{l}_{Rx}}\times 100\% $$

$$ HI=\frac{D_2-{D}_{98}}{D_{Rx}}\times 100\% $$

where Vol_Target_ and Vol_Rx_ are the PTV volume and the volume receiving the prescription dose, respectively, and D_Rx_ is the prescribed dose to the target. The ideal CI for a plan is 1, and HI should be as small as possible.

In addition to the mean dose for each OAR, the lung V_5_, percentage of lung volume receiving above 5 Gy, was reported.

### Quality assurance

An ionization chamber array (MatriXX, IBA Dosimetry, Bartlett, TN) was used to measure the dose distribution in a slab phantom (MultiCube) for validating the treatment plans in this study. The dimensions of MultiCube are 31.4 cm (L) x 34 cm (W) x 34 cm (H), and the distance from the detector plane to the top surface is 11 cm. It was used to measure only the dose distribution at one end of PTV. The electronic circuit part of MatriXX must be kept away from direct irradiation to avoid being damaged. Since none of the commercial QA devices is big enough to cover the high dose region for CSI, the dose distribution at the beam junctions was verified by EBT2 radiochromic film (International Speciality Products, Wayne, NJ) placed in a water-equivalent slab phantom, as shown in Additional file 2: Figure S2. The dimensions of the phantom for film measurement were 90 cm (L) x 30 cm (W) x 20 cm (H). The film was placed at the depth of 10 cm. For the film measurement, the phantom was large enough to encompass the dose calculation zone of the QA plans.

Setup errors were simulated by shifting the lower isocenter with ±1, ±3, ±5 mm for the plan with all 0° collimator angle which represent the worst case scenario at the beam junctions. In addition to the set of 6 collimator combinations, a few plans with 5° collimator rotation were generated for assessing its effects on blurring the dose in the field junctions.

## Results

The planning parameters for the six plans and resulted dose distribution parameters for the PTV and OARs are shown in Table 4. There were no significant differences among the mean doses for the PTV in all plans, and the OAR doses, in general do not pose a concern. Also there were no meaningful differences in HI, CI, the numbers of segments, MUs and delivery times for the plans with 0° and 30° collimator angles. However, for the plans with 45° collimator angle, the increased number of beams led to increased numbers of segments, MUs and the delivery time. The mean HI and CI values were slightly lower than that of the plans with 0° and 30° collimator angles. The plan PAc45c45 had the highest numbers of segments, MUs and the longest delivery time. The mean CIs for PTV2 were significantly lower than that for the whole PTV.

**Table 4:** Dose statistics for the PTV and OARs, and treatment planning parameters for plans

Target	Parameters	PAc0c0	PAc0c30	PAc30c30	PAc0c45	PAc30c45	PAc45c45
PTV	Mean (cGy)	3753.2 ± 44.8	3721.5 ± 60.2	3718.6 ± 38.8	3738.5 ± 32.9	3741.4 ± 55.7	3735.5 ± 31.4
	D95 % (cGy)	3600	3600	3600	3600	3600	3600
	D2 % (cGy)	3908.6.2 ± 41.2	3936.4 ± 35.2	3937.1 ± 39.8	3928.1 ± 47.3	3941.1 ± 51.7	3951.1 ± 56.6
	D98 % (cGy)	3520.3 ± 22.5	3528.4 ± 28.9	3538.3 ± 26.4	3529 ± 21.7	3518.2 ± 25.7	3532 ± 20.2
	V107 %	8.9 ± 2.5 %	9.1 ± 3.2 %	9 ± 3.5 %	9.2 ± 3.4 %	9.1 ± 2.8 %	9.3 ± 3.7 %
Lenses	Mean (Gy)	6.93 ± 0.41	6.88 ± 0.45	7.08 ± 0.55	6.95 ± 0.57	6.96 ± 0.82	7.11 ± 0.63
Optic nerves	Mean (Gy)	21.2 ± 2.76	21.4 ± 2.26	21.2 ± 2.55	22.1 ± 2.89	21.3 ± 2.68	21.9 ± 3.11
Parotids	Mean (Gy)	12.66 ± 1.82	13.15 ± 1.97	12.16 ± 1.88	13.06 ± 1.9	13.4 ± 1.72	13.27 ± 1.43
Thyroid	Mean (Gy)	12.88 ± 2.19	12.91 ± 2.37	12.42 ± 2.84	12.28 ± 2.41	13.44 ± 2.77	13.05 ± 2.76
Lungs	V5 %	42.1 ± 7.66 %	42.8 ± 7.52 %	43.6 ± 8.58 %	44.2 ± 8.64 %	45.1 ± 8.09 %	44.7 ± 8.60 %
	Mean (Gy)	6.24 ± 0.52	6.46 ± 0.78	6.41 ± 0.83	6.38 ± 0.77	6.33 ± 0.29	6.51 ± 0.44
Esophagus	Mean (Gy)	15.1 ± 2.9	15.7 ± 2.2	15.5 ± 2.36	14.8 ± 2	15.3 ± 2.86	15.8 ± 2.11
Heart	Mean (Gy)	6.3 ± 1.58	6.1 ± 2.05	6.1 ± 2.95	6.5 ± 2.5	6.4 ± 2.8	6.2 ± 2.34
Liver	Mean (Gy)	5.31 ± 0.52	5.22 ± 0.88	5.45 ± 0.85	5.36 ± 0.49	5.1 ± 0.73	5.62 ± 0.76
Kidneys	Mean (Gy)	4.85 ± 0.65	4.92 ± 0.74	4.9 ± 0.53	5.02 ± 0.81	4.92 ± 0.69	5.23 ± 0.8
Planning parameters							
Number of arc beams		4	4	4	7	7	7
Segments		236.9 ± 8.2	239.3 ± 6.4	241.7 ± 6.3	278.7 ± 5.3	277.4 ± 7.6	285.3 ± 8.8
Mus		1033.6 ± 55.3	1064.1 ± 39.8	1028.5 ± 61.4	1316.3 ± 78.1	1335.4 ± 56.7	1393.8 ± 51.4
Delivery time (s)^*^		233.9 ± 16.4	236.3 ± 13.5	239.2 ± 15.7	310.7 ± 25.6	313.2 ± 18.1	323.9 ± 15.8
HI		10.96 ± 1.6 %	10.99 ± 1.4 %	11.05 ± 1.7 %	11.25 ± 1.6 %	11.2 ± 2.9 %	11.58 ± 1.9 %
CI (whole PTV)		90.6 ± 2 %	90.5 ± 2.4 %	90.1 ± 2.4 %	89.1 ± 3.1 %	88.6 ± 2.9 %	87.5 ± 1.9 %
CI (Spine only)		69.2 ± 3 %	68.5 ± 3.3 %	68.6 ± 3 %	67.2 ± 2.1 %	65.8 ± 2.4 %	66.1 ± 2.3 %

* Delivery time: included the beam-on, the gantry and collimator rotation between arc beams, not the shift between isocenters

**Fig. 2:**
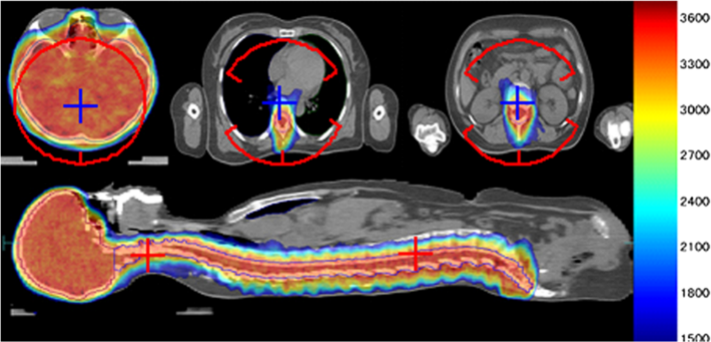
Dose distributions in selected transversal and sagittal planes for a patient (*PlanAxesse*). Color wash range: 1500-3750cGy (Maximum dose: 3952.6cGy); red crosses and arcs: beam isocenters and arc beams; blue crosses: points on the axis through two beam isocenters

The dose distributions in selected sagittal and transversal planes for one patient are shown in Fig. 2. The dose distribution was somewhat elongated in the anteroposterior direction.

**Fig. 3:**
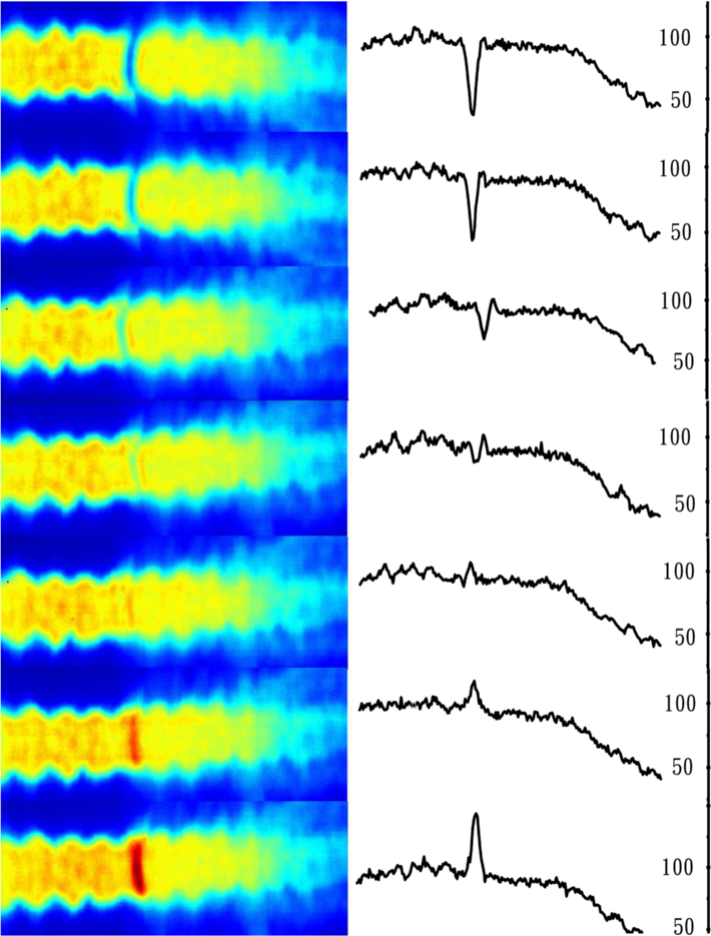
Dose distributions by film measurement at the junction region of arc beams with two isocenters. The collimator angles were at 0°. The dose was normalized to 180cGy. From top to bottom: the Y coordinate of the lower isocenter was shifted −5, −3, −1, 0, 1, 3, 5 mm, respectively

**Fig. 4:**
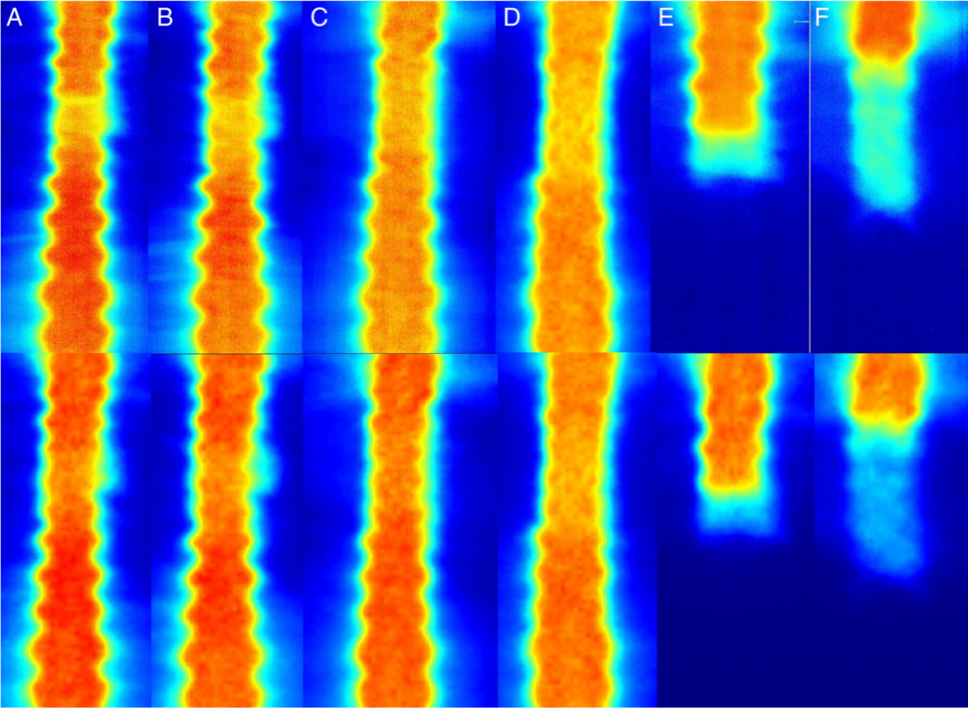
Dose distributions in the junction regions of arc beams with different isocenters and collimator angles. The upper and lower parts were the measured (by films) and calculated dose distributions, respectively. From Part **a** to Part **f**: the collimator angles of the upper and lower arc beams were 0°/0°, 0°/5°, 30°/30°, 45°/45°, 0°/NA, 30°/NA, respectively. The Part **e** and Part **f** were the dose distributions of the beams with the upper isocenter. The dose was normalized to 180cGy

Figure 3 showed pronounced dose difference as the longitudinal shift of the lower isocenter reached 3 mm and greater. Furthermore, there was roughly a 1 mm lower dose gap between the beams with different isocenters. This gap disappeared when the collimator angle was off 0° for at least one set of beams, as seen in Fig. 4. The dose distributions in the beam overlapping region were exhibited in Fig. 4e and f. The beam penumbras were enlarged significantly in the overlapping region.

## Discussion

As previously reported by a few studies on the application of VMAT for CSI [2–4], VMAT showed advantages in improved dose conformity and homogeneity, as well as fast treatment delivery, compared to conventional non-IMRT techniques. The difficulty in field junction matching was resolved by inverse planning with a modern TPS automatically and reliably accurate dose calculation.

However, there is still a need for a consented common clinical or planning guidelines for CSI using VMAT. In the study by Fogliata et al. [3], five patients underwent CSI were treated in 5 different institutes. Two of them were supine and the others were prone, and the immobilization techniques were also different. The plan optimization technique also varied. In addition, three sets of beams with different isocenters were used for 3 patients, even though the maximum target length was only 78.4 cm.

Undoubtedly, patients will be more comfortable in supine position than prone. The thermoplastic mask, knee support and vacuum bag for the body can all contribute to improving patient immobilization and therefore target localization, especially for a long target in CSI.

A beam with 45° collimator angle can cover a 48.5 cm long target. Thus even a target of 90 cm long in CSI can be treated by beams with two isocenters. Table 4 indicates that there was little difference in plan quality among the plans with different combinations of beam collimator angles. Two sets of beams with 30° collimator angle were enough for the 80.7 cm long target. This only involved one field junction, and the couch moved only once during the treatment. Using two beam isocenters avoided added uncertainties associated with one more beam setup.

Figure 3 showed the dose distributions in the junction region when the collimator angle of the beams was at 0°. A 3 mm longitudinal error of the lower isocenter led to more than 20 % difference in the dose distribution. The middle three distributions corresponding to 0 and ±1 mm isocenter shifts were all acceptable. The typical mechanical accuracy for couch position with a modern linac is on the order of 1 mm [4]. So with reliable patient immobilization the dose errors in the junction region could be well within clinical tolerance. It is interesting to observe a small dose gap around 3 % when there was no artificially introduced setup error, as shown by the middle plot in Fig. 3. There are several reasons for the low dose gap, including the couch position error, and the beam model for a large field penumbra may not be perfect as the modeling of jaw transmission also comes into play, especially when the collimator angle is set to 0°. The dose gap was revealed by film measurement, which provided a high spatial resolution. The gap was not seen in the TPS calculated dose distribution. A fast X-ray voxel Monte Carlo algorithm [12–14] was adopted in the TPS but it still took over 5 h with a dose grid of 3 mm × 3 mm × 3 mm. Using a finer dose grid with our current hardware was not practical for such a large target volume.

The dose distribution in the field junction region with different combinations of the beam collimator angles are shown in Fig. 4. It was interesting that the aforementioned gap disappeared as the collimator angle was not zero for at least one set of beams. Even 5° collimator angle was enough to make the gap to disappear. The major reason for this might be due to the broadened beam penumbra when the collimator angle was off 0°. When the collimator angle was up to 30° and more, the overlap regions between fields were expanded substantially. For example, with 35 cm separation between two isocenters, the field overlaps were about 2.5 cm and 4 cm for 0°/0° and 30°/30° collimator angle combinations, respectively. The beam penumbras were clearly broadened in the overlapping regions (Fig. 4e, f). Similar results were also found by Chen [4]. In his study, the collimator angles of the upper and lower arc beams were 5° and 355°, respectively. A longitudinal shift of ±3 mm for the lower isocenter led to less than 15 % dose errors in the junction region. The dose distribution at the field junction may also be dependent on the different linear accelerators or different TPS used.

Although VMAT is known to offer advantageous dose distribution over conventional three-dimensional conformal radiotherapy and IMRT [2], one should recognize an increased volume of healthy tissue being irradiated with VMAT. We used three partial arcs with the lower isocenter in order to reduce the volume of body irradiated. As a consequence the dose distribution was slightly elongated in the anteroposterior direction (Fig. 2). Overall, the results of this study, including the patient whose target was 80.7 cm long, were compatible to those reported in other studies, but all our patients were treated with only two beam isocenters. An increased numbers of segments, MUs and delivery time in the plans with 45° beam collimator angle were reasonable, considering the large target volume and the alternative of using three beam isocenters.

## Conclusion

A highly conformal and homogeneous VMAT planning technique for CSI using an Axesse® linear accelerator was developed. By setting the collimator angles to 45°, only two beam isocenters were needed for treating a target with length up to 90 cm. The plan quality was basically same, regardless of the beam collimator angle used. Setting the collimator angle off 0° for at least one set of beams could make the plan less susceptible to localization error of the beam isocenter.

## Additional files

Additional file 1: Figure S1. A 40 cm × 40 cm field with a 45° collimator angle. (TIF 15 kb). Additional file 2: Figure S2. The sketch of dose distribution verification in fields’ junction with films. The film was placed in the middle of square, slab phantoms, which was placed in the middle of two arc beam isocenters. The distance of two isocenters was 350 mm. (JPG 39 kb).

## Abbreviations

3D CRT: three-dimensional conformal RT
CI: conformity index
CSI: craniospinal irradiation
CTV: clinical target volume
DLG: dynamic leaf guides
DVH: dose-volume histogram
HI: homogeneity index
IMRT: intensity modulated radiation therapy
MLC: multileaf collimator
OAR: organ-at-risk
PTV: planning target volume
QA: quality assurance
TPS: treatment planning system
V_5_ for lung: the percentage lung volume receiving 5 Gy
VMAT: volumetric modulated arc therapy
