# Supplementary figures and images for: Collimator rotation in volumetric modulated arc therapy for craniospinal irradiation and the dose distribution in the beam junction region
Source: Radiat Oncol. 2015 Nov 19;10:235. doi: 10.1186/s13014-015-0544-z (PMC4653929; doi:10.1186/s13014-015-0544-z)

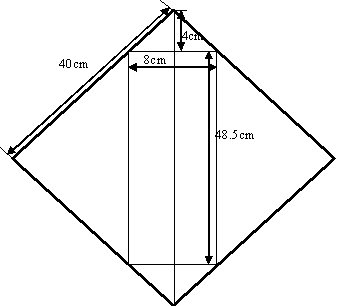

Supplement: Additional file 1: Figure S1. — A 40 cm × 40 cm field with a 45° collimator angle. (TIF 15 kb). [file 13014_2015_544_MOESM1_ESM.tif]

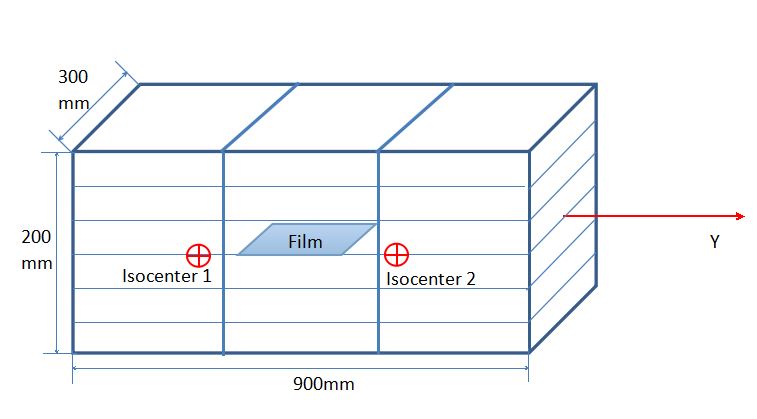

Supplement: Additional file 2: Figure S2. — The sketch of dose distribution verification in fields’ junction with films. The film was placed in the middle of square, slab phantoms, which was placed in the middle of two arc beam isocenters. The distance of two isocenters was 350 mm. (JPG 39 kb). [file 13014_2015_544_MOESM2_ESM.jpg]
